# Turbulence of glutamine metabolism in pan-cancer prognosis and immune microenvironment

**DOI:** 10.3389/fonc.2022.1064127

**Published:** 2022-12-07

**Authors:** Songjiang He, Shi Zhang, Yi Yao, Bin Xu, Zhili Niu, Fuben Liao, Jie Wu, Qibin Song, Minglun Li, Zheming Liu

**Affiliations:** ^1^ Cancer Center, Renmin Hospital of Wuhan University, Wuhan, China; ^2^ Department of Anesthesiology, Renmin Hospital of Wuhan University, Wuhan, China; ^3^ Department of Clinical Laboratory, Renmin Hospital of Wuhan University, Wuhan, China; ^4^ Department of Radiation Oncology, University Hospital, Ludwig- Maximilians-Universität München (LMU), Munich, Germany

**Keywords:** glutamine metabolism, prognosis, cell cycle, tumor microenvironment, immunotherapy

## Abstract

**Introduction:**

Glutamine is characterized as the nutrient required in tumor cells. The study based on glutamine metabolism aimed to develop a new predictive factor for pan-cancer prognostic and therapeutic analyses and to explore the mechanisms underlying the development of cancer.

**Methods:**

The RNA-sequence data retrieved from TCGA, ICGC, GEO, and CGGA databases were applied to train and further validate our signature. Single-cell RNA transcriptome data from GEO were used to investigate the correlation between glutamine metabolism and cell cycle progression. A series of bioinformatics and machine learning approaches were applied to accomplish the statistical analyses in this study.

**Results:**

As an individual risk factor, our signature could predict the overall survival (OS) and immunotherapy responses of patients in the pan-cancer analysis. The nomogram model combined several clinicopathological features, provided the GMscore, a readable measurement to clinically predict the probability of OS and improve the predictive capacity of GMscore. While analyzing the correlations between glutamine metabolism and malignant features of the tumor, we observed that the accumulation of TP53 inactivation might underlie glutamine metabolism with cell cycle progression in cancer. Supposedly, CAD and its upstream genes in glutamine metabolism would be potential targets in the therapy of patients with IDH-mutated glioma. Immune infiltration and sensitivity to anti-cancer drugs have been confirmed in the high-risk group.

**Discussion:**

In summary, glutamine metabolism is significant to the clinical outcomes of patients with pan-cancer and is tightly associated with several hallmarks of a malignant tumor.

## Introduction

The precise control of cellular metabolic processes maintains the morphology of the cells (1–3), but during transformation into the malignant state, the cells acquire a series of hallmarks, including metabolic reprogramming (4). The survival of tumor cells depends on the intake of large amounts of nutrients. Therefore, metabolic reprogramming is effectuated through alterations in several signaling pathways to maintain the unlimited proliferative capacity of tumor cells (2–5). Since Otto Warburg explicated aerobic glycolysis (6), glucose metabolism has been the center of tumor metabolic research, while only a few studies have focused on other nutrients, such as glutamine. However, a recent study by Bradley et al. (7) reported that the tumor microenvironment (TME) comprises the most sugar-consuming population in tumor-associated macrophages (TAMs), which are primarily dependent on sugar metabolism for energy supply. However, tumor cells show a significant preference for glutamine metabolism. This finding has greatly shaken the role of carbohydrate metabolism in tumor metabolism in recent decades.

Glutamine is the most abundant free amino acid in circulation (8) and is used as a ready source of carbon and nitrogen to support biosynthesis, energy metabolism, and intracellular homeostasis of tumor cells (9). Under the catalysis of glutamine-specific glutaminase (GLS), glutamine is taken into cells *via* the glutamine transporter protein ASCT2 (also known as SLC38A5) and SN2 and is catabolized to glutamate; the increased expression of this gene is essential for the development of cancer (10). Subsequently, glutamine is further catabolized into α-Ketoglutaric acid (α-KG) by glutamine dehydrogenase (GLUD), and a group of transaminases (including GOT, GPT, and PSAT) enters the tricarboxylic acid (TCA) cycle, which provides energy for cell growth. Then, glutamine and its metabolites support the synthesis of biomolecules, such as nucleic acids, proteins, and fatty acids (11). In addition, glutamine plays a crucial role in cellular autophagy, reactive oxygen species (ROS) stress, and the formation of tumor microenvironment (7, 12, 13). Owing to the non-negligible role of glutamine on cellular neo-metabolism, several studies (14–16) have shown that glutamine deprivation leads to tumor cell death.

In the present study, a series of bioinformatics and machine learning approaches have been applied to investigate the potential impact of glutamine metabolism on patient prognosis, immune status, and treatment outcome in pan-cancer analysis and identify critical pathways and genes involved in the process of glutamine metabolism. The resulting specific glutamine metabolism-related genes were used to construct a prognostic glutamine metabolism-related risk score (GMscore) and predict the overall survival (OS) of patients. The GMscore demonstrated stable and accurate predictive capability than conventional clinical features. Next, we constructed a scoring nomogram as a survival prediction model to further improve the prediction accuracy. In addition, we observed significant associations between glutamine metabolism and cell cycle processes, and CAD was significantly associated with prognosis in glioma patients with IDH1 mutations. We also evaluated the immune infiltration of patients in both risk cohorts and provided valuable hints for anti-cancer drug and immune checkpoint blockade (ICB) decisions based on glutamine metabolism genes.

## Materials and methods

### Data acquisition and preprocessing

Publicly available transcriptome data with the matching clinical annotation obtained from the Cancer Genome Atlas program (TCGA, https://portal.gdc.cancer.gov/) were utilized as discovery and validation cohorts, respectively. We randomly categorized 70% of the 9370 (6559/9370) patients involved in 32 types of tumors, without samples of acute myeloid leukemia (LAML), as the training set, and the remaining (2811/9370) comprised the testing set. Additionally, 899 samples from the International Cancer Genome Consortium (ICGC, https://dcc.icgc.org/), four individual microarray datasets [GSE21653 (n=252), GSE72094 (n=398), GSE17674 (n=44), GSE2748 (n=28)] form Gene Expression Omnibus (GEO, https://www.ncbi.nlm.nih.gov/geo/), and two individual RNA-sequence datasets including 325 and 693 samples from Chinese Glioma Genome Atlas (CGGA, http://www.cgga.org.cn/) were retrieved for further validation. We also obtained 7862 normal samples in the Genotype-Tissue Expression (GTEx) from USCS Xena (http://xenabroswer.net/hub). As reported previously (17–19), all the microarray and RNA-sequence data were normalized to transcripts per million (TPM) values and log2 transformed. The gene sets of hallmarks of cancer were retrieved from the Molecular Signatures Database (MSigDB, https://www.gsea-msigdb.org/gsea/msigdb/), and the gene sets related to immune infiltration were obtained from a previous study by Charoentong et al. (20). Somatic mutation data of the 32 types of tumors sorted in the mutation annotation format (MAF) files had been analyzed using the R package “maftools.” RNA-sequence data of the glutamine metabolism inhibition and the 24 patients who received anti-PD1 therapy were retrieved from GEO [GSE120345 (n=10), GSE115821(n=24)] and normalized into TPM values.

### Establishment of the prognostic GMscore

Glutamine metabolism engages in many biological pathways, including the TCA cycle, biosynthesis, TME formation, autophagy, ROS, and signal transduction. Consequently, we combined the results of several latest reports (9, 21–26) and gene sets of glutamine metabolism from MsigDB (27). As a result, 118 genes (**Supplemental Table 1**) converged as the initial biomarkers of glutamine metabolism for signature training.

Subsequently, the R package “coxph” was used to calculate the HR value and p-value for each gene involved in the initial biomarkers to assess the correlations of the expressions of the 118 genes in the OS of patients in the TCGA training set. Subsequently, 67 candidates, with a threshold of p-value<0.001, were entered into the least absolute shrinkage and selection operator (LASSO) Cox regression model *via* the action of a penalty parameter (λ) to the Cox regression model that led to zero coefficients. In our studies, 25 genes retained their coefficients with an optimal λ after LASSO regularization. Furthermore, Mantel test was used to identify genes that have the same expression mode. After excluding such genes, a GMscore for each sample, based on the expression of the last 21 genes, was calculated as follows:


$$GMscore=\sum_{i=1}^{n}Coefficients\left(mRNAi\right)\;\times \;Expression\left(mRNAi\right)$$

### Survival analysis

The patients were divided into two risk groups, GMscore-high and GMscore-low cohorts, respectively, based on the median value of GMscore. Then, the Kaplan–Meier method was used to plot the survival curves, and the log-rank test was applied to estimate the differences in the prognosis between the two risk cohorts. Multivariate Cox regression analysis was used to assess the risk significance for survival. Moreover, R package “survConcordance”, a time-dependent concordance index (C-index) was used to compare the predictive probability among different variables.

### Construction of comprehensive prognostic models

A comprehensive scoring nomogram was generated to improve the predictive capacity for survival *via* a combined GMscore with detailed clinicopathological features, including gender, age, stage, and cancer types. In addition, calibration curves for predictions of 1-, 3-, and 5-year OS were plotted to compare with the actual OS. Using the R package “TimeRoc”, time-dependent receiver operating characteristic (tROC) analysis was conducted to estimate the accuracy of the nomogram and compare the predictive capacity among different variables.

### Single cells analysis

The RNA-sequence data (read counts and TPM value) of 1067 single cells, which included cells in different cell cycles retrieved from the GEO database (GSE146773). Then, pseudo time trajectory analysis was performed to speculate the development correlation of the clusters with R package “monocle2”, and plot the expressions of genes engaged in glutamine metabolism against the development of cell cycle progress.

### Immune infiltration analysis

Gene set enrichment analysis (GSEA), accomplished with an R package “clusterProfiler”, was used to enrich the genes highly regulated in the GMscore-high cohort. Four algorithms, named ESTIMATE (28), xCell (29), CIBERSORT (30), and Tumor Immune Estimation Resource (TIMER, cistrome.shinyapps.io/timer) (31), were used to measure the absolute and relative abundance of immune cells between the different cohorts. In addition, the tumor purity of each sample was assessed by the ESTIMATE algorithm. Cytolytic activity (CYT) score was defined as the geometric mean of PRF1 and GZMA (32).

### Additional bioinformatic and statistical analyses

Based on the requirements of R package, R (v.4.2.1 and v.4.0.0, http://www.r-project.org) was applied for all statistical analyses. The Mantel test was used to evaluate the correlations among the expressions of genes using the R package “linkET”. The R package “clusterProfiler” was applied to complete the enrichment analysis. Protein-protein interaction (PPI) network was constructed using STRING database and “Cytoscape” software. Based on the transcriptome data (TPM value) from the TCGA training set, z-scores of initial biomarkers and five gene sets of hallmarks of cancer were calculated by the “z score” algorithm provided by R package “GSVA”. The R package “DESeq2” was utilized to identify the differential genes between the two cohorts with RNA-sequence data (read counts). The R package “pRRophetic” was used to reckon the sensitivities of commonly used chemotherapeutic drugs and target epidermal growth factor receptor (EGFR) drugs of each patient. The responses to ICB therapy of samples from TCGA were predicted by the TIDE algorithm (33). Then, the R package “randomForest”, a random forest algorithm was applied to screen the critical candidates related to the prediction of ICB therapy responses among initial biomarkers. The R package “pROC” was used to plot the ROC curves and calculate the area under the curve (AUC) to evaluate the feasibility of the model. p-value<0.05 was considered statistically significant.

## Results

### Identification of a set of 21 glutamine metabolism-associated genes

**Figure 1:**
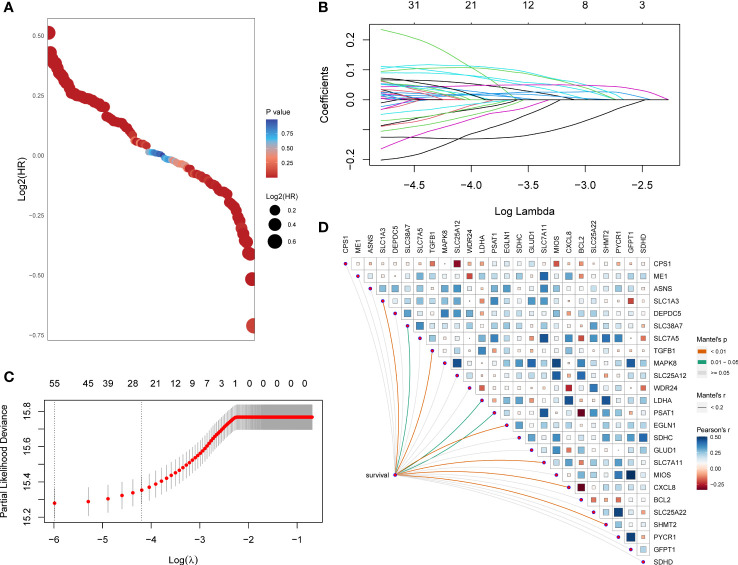
A set of 21 glutamine metabolism-related genes was identified. **(A)** Exhibition of HR and p-values of 118 genes calculated by univariate Cox regression analysis. **(B)** 67 genes entered into the LASSO Cox proportional risk model with a threshold of p-value<0.001. **(C)** 25 genes with their coefficients were filtered with the optimal λ. **(D)** Correlations of expression level among 25 candidates and the genes related to survival were assessed using the Mantel test.

First, the HR and p-values of each gene involved in the 118 initial biomarkers were calculated based on the transcriptome data with the matching clinical annotation of the TCGA pan-cancer training set (**Figure 1A**). After applying a filtering threshold of p-value<0.001, 67 candidates were entered into LASSO logistic regression analysis **(** **Figure 1B**). A ten-fold cross-validation was used to overcome the over-fitting effect (**Figure 1C**), confirming an optimal λ value of 0.01507538; finally, 25 glutamine metabolism-related genes retained their coefficients. Subsequently, a Mantel test was conducted on the 25 genes (**Figure 1D**), and *SLC1A3*, *SLC38A7*, *TGFB1*, *LDHA*, *PSAT1*, *EGLN1*, *SLC7A11*, *CXCL8*, and *SHMT2* showed a significant correlation with patients’ OS. We also observed high correlation coefficients between *MIOS* and *GFPT1*, *SLC25A12* and *MAPK8*, *PYCR1* and *SLC25A22*, *LDHA* and *CXCL8*, and *MIOS* and *MAPK8*. To overcome the over-fitting effect caused by a large number of samples in pan-cancer analysis, we randomly removed one of the paired genes (*MIOS*, *SLC25A12*, *PYCR1*, and *LDHA*) and further analyzed the 21 glutamine metabolism-related genes.

### Establishment and validation of GMscore

**Figure 2:**
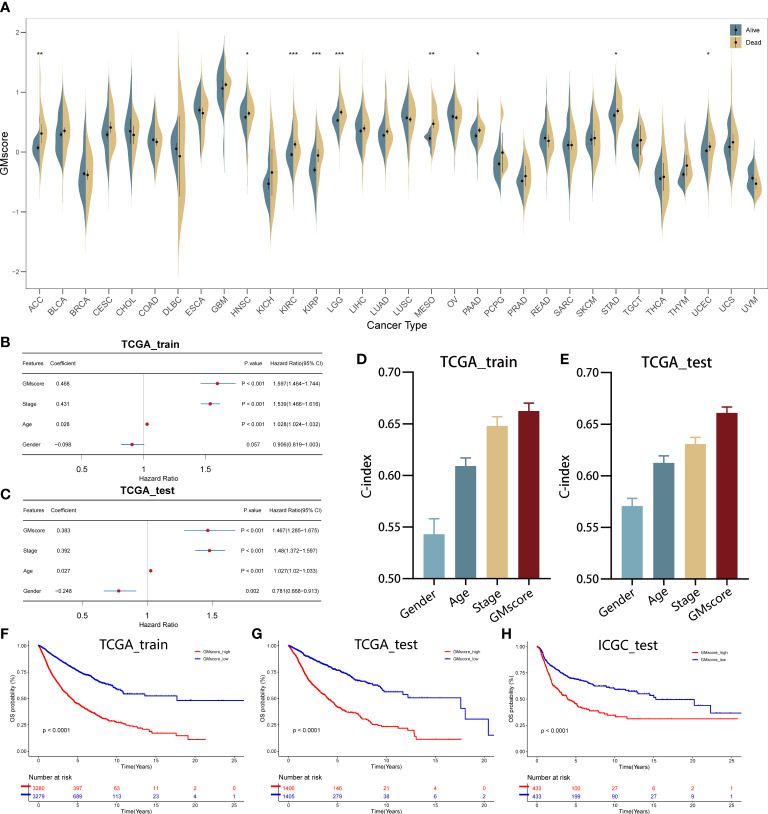
Establishment and validation of the GMscore in pan-cancer. **(A)** Distribution of GMscore among 32 types of cancers. *p < 0.05; **p < 0.01; ***p < 0.001. **(B, C)** Multivariate Cox regression demonstrated that GMscore is an independent risk factor among all variables in the TCGA training and test cohorts. **(D, E)** The c-index of GMscore ranked first among all parameters in both cohorts. **(F–H)** Kaplan-Meier analysis was used to validate patients with low GMscore who showed improved prognosis in the TCGA training and test cohorts and ICGC cohort, respectively.

A total of 21 genes were identified and used to establish a signature to predict the OS of the pan-cancer patients based on their coefficients and expression levels (TPM value). Thus, a GMscore for each sample was calculated *via* the established formula mentioned before. While comparing the GMscores among the 32 types of tumors, we observed that the tumors originating from the brain, such as GBM and LGG exhibited the highest GMscore; also, distinct disparities were observed between dead patients and those who were still alive (**Figure 2A**). Then, multivariate Cox regression analysis was performed on four variables, including GMscore (continuous value), gender (male or female), age (continuous value), and stage (I–IV), and the results demonstrated that the GMscore was an independent risk factor among all the variables (p<0.001) in TCGA training set for validating the effectiveness of the signature (**Figure 2B**). The results were validated in the TCGA test cohort (**Figure 2C**). Furthermore, to measure the predictive capacity of GMscore, we compared the C-index of the four variables, and the results showed that the GMscore ranked first among all the variables in the training set (**Figure 2D**); a similar conclusion was obtained in the TCGA test cohort (**Figure 2E**). Next, we divided the training set samples into two risk groups according to the median value of GMscore. Kaplan–Meier analysis proved that patients with high GMscore had poor OS in the training set (p<0.001, **Figure 2F**) and had been verified in the test and external validation sets from ICGC, respectively (p<0.001, **Figure 2G, H**). Moreover, to prove that the GMscore could be effective in specific tumors, Kaplan–Meier analysis was conducted on 32 types of tumors independently. Consequently, we exhibited the top four cancer types (KIRC, LUAD, MESO, and UCEC) that showed significant differential prognosis between different risk cohorts (**Supplementary Figures 1A–D**). Four individual datasets retrieved from GEO demonstrated the correctness of the result (**Supplementary Figures 1E–H**). In addition, RNA-sequence data from CGGA, collected from a large number of glioma patients, had been used for further validation. As expected, distinct differences were detected in the OS between the two risk groups (**Supplementary Figures 1I, J**). It was worth noting that there was a distinct difference in the baseline when we compared the GMscore of sequence data retrieved from TCGA and CGGA, respectively (**Supplementary Figure 1K**), indicating that differences in our dietaries or ethnicities might have a great effect on GMscore. Therefore, defining ethnic origin should be better concerned when we are conducting patient risk assessments in clinics. In summary, these results revealed that GMscore was an individual risk factor distinct from other clinicopathological features but an ideal model with a better predictive probability of prognosis compared to these features.

### Construction of an integrated model for predicting the OS of pan-cancer

**Figure 3:**
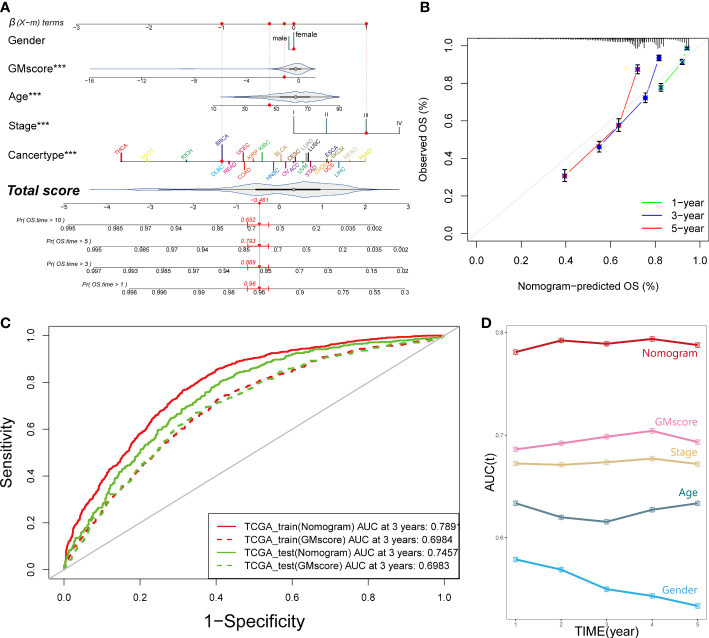
An integrated prognostic model was constructed to improve the estimation of survival probability for pan-cancer. **(A)** A comprehensive nomogram was generated to predict the OS possibilities at 1, 3, 5, and 10 years. ***p < 0.001. **(B)** Calibration curves of 1-year (green dotted line), 3-year (blue dotted line), and 5-year (red dotted line) prediction were close to the ideal performance (45°, grey line). OS, overall survival. **(C)** Predictive efficiency of the nomogram signature was better than GMscore in the TCGA training and test cohorts. **(D)** Time-dependent ROC analysis provided a robust capability to predict the OS probability compared to the other conventional characters.

In order to equip GMscore with an excellent predictive capacity and provide an individual risk assessment for each patient, we established an integrated scoring nomogram by combining the GMscore with other clinicopathological features, including gender (male or female), age (continuous value), stage (I–IV), and cancer types of patients (**Figure 3A**). This action led to a readable and quantitative measurement for the GMscore to clinically predict the probability of adverse events that could be provided for each sample. The calibration curves were plotted to confirm the accuracy of the comprehensive model (**Figure 3B**). The results showed that the predictions of 1-year (green dotted line), 3-year (blue dotted line), and 5-year (red dotted line) OS were close to the ideal performance (45° line), suggesting that the correction of the model could be well-described. tROC analysis compared the predictive probability of nomograms with GMscore alone, which shows that the predictive capacity of nomograms was always higher than the GMscores in both the training and test sets (**Figure 3C**). Furthermore, compared to the other four variables, the nomogram exhibited the highest prediction of OS (**Figure 3D**). As a result, we concluded that combining GMscore with conventional clinicopathological features played a critical role in the clinical assessment of patients.

### GMscore was significantly correlated with cell cycle progression

**Figure 4:**
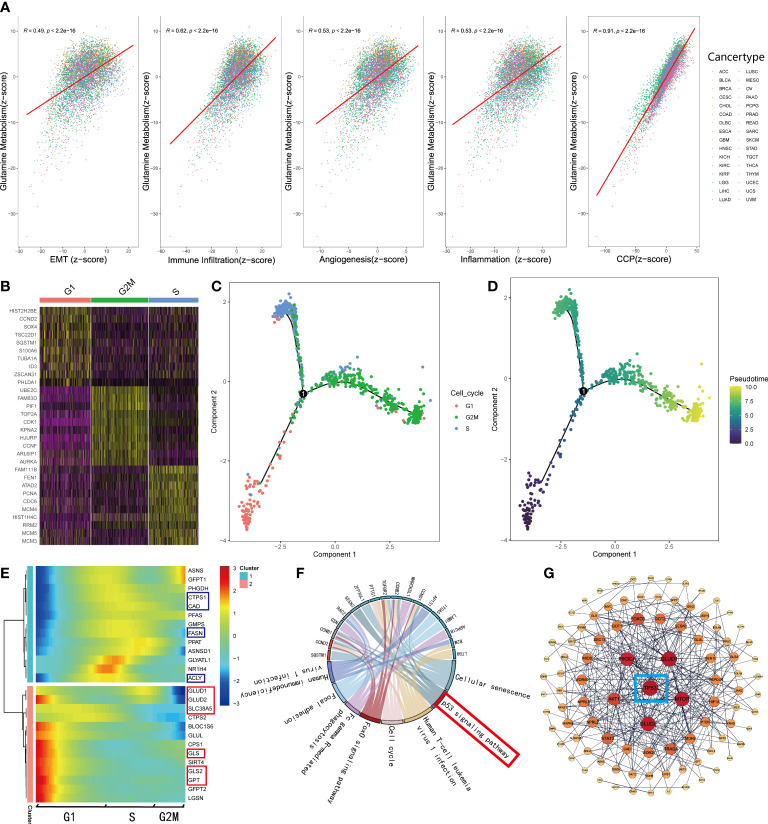
GMscore was significantly correlated with cell cycle progression. **(A)** Correlations between glutamine metabolism and hallmarks of the malignant tumor. **(B)** Differential genes among G1, S, and G2 phases. **(C)** Pseudo time trajectory analysis conjectured the developmental correlations of the clusters based on the differential genes. **(D)** Pseudo time trajectory analysis exhibited the developmental direction of the three clusters. **(E)** The expression level of genes involved in glutamine metabolism was enriched in two distinctive clusters. **(F)** Significantly upregulated genes in the G1 phase were enriched in the p53 signaling pathway. **(G)** PPI network revealed the critical gene regulating the metabolism of glutamine: TP53.

As mentioned before, normal cells acquire a series of hallmarks of the malignant tumor during the process of transforming into a malignant state (4). Recent studies (7, 34–36) illustrated that the process of glutamine metabolism and the enzymes involved play a significant role in many biological pathways, including angiogenesis, immune infiltration, epithelial-mesenchymal transition (EMT), and mitosis. Thus, to investigate the connections between glutamine metabolism and the hallmarks of malignant tumors, we searched the gene sets of EMT, angiogenesis, response to inflammation, and cell cycle procession (CCP) from MSigDB. Additionally, the gene sets related to immune infiltration were retrieved from a study by Charoentong et al. (20). We qualified all the features, including EMT, angiogenesis, inflammation, immune infiltration, CCP, and glutamine metabolism for each sample using the z-score algorithm. Subsequently, we calculated the Pearson’s correlation coefficient (r) between glutamine metabolism and these malignant features, respectively, and showed significant correlations between glutamine metabolism and malignant features (p<0.001); the strongest correlation was observed between glutamine metabolism and CCP (R=0.91, p<0.001, **Figure 4A**). Interestingly, only a few studies have been reported on glutamine metabolism and CCP in recent years. To explore how these correlations are distributed, we observed the associations in various cancer types. The results demonstrated that a strong correlation was retained in almost all tumor types since R>0.9 (p<0.001, **Supplementary Figure 2A**). Moreover, we also investigated the correlations between glutamine metabolism and the other four features successively, and the top eight cancer types with the highest value were presented for each feature (**Supplementary Figures 2B–E**).

To elucidate the correlation between glutamine metabolism and CCP, we retrieved single-cell RNA-sequence data from 346 cells in the G1 phase, 334 cells in the S phase, and 387 cells in G2M phase (37). Based on the differential genes among the three clusters (**Figure 4B**), we conducted a pseudo time trajectory analysis to speculate on the developmental correlations among the three clusters (**Figure 4C**, **Supplementary Figure 3A**) and found that these cells were divided into three clusters at different states (**Supplementary Figure 3B**); this finding supported the significance of our study. According to the chronological order of development among clusters (**Figure 4D**), we plotted the expressions of the genes involved in the glutamine metabolism processes, downloaded from MSigDB (**Figure 4E**). The heatmap illustrated that the genes involved in decomposing glutamine to enter the TCA cycle gathered in cluster 2, while those engaged in the biosynthesis were enriched in cluster 1, which correspond to the G1 and S phases, respectively. Next, we selected the genes at key points of decomposing glutamine and observed their change in expressions along with development. Interestingly, the genes involved in glutamine metabolism are highly expressed only in the G1 phase (**Supplementary Figures 3C–F**). Furthermore, the RNA-sequence data of glutamine inhibitor-treated mice cell lines retrieved from GEO showed that the expression levels of these genes (*Asns, Gfpt1, Ctps1, Cad, Pfas, Gmps, Fasn, Ppat, Asnsd1, Glyatl1, Nr1h4, Acly, Glud1, Ctps2, Bloc1s6, Glul, Cps1, Gls, Gls2, Gfpt2, Lgsn*) were down-regulated obviously (**Supplementary Figure 3G**). Based on these results, we supposed that cells uptake abundant glutamine in the G1 phase to produce substantial energy to satisfy the biosynthesis and mitosis, and a part of glutamine and metabolites participate in the process of biosynthesis during the S phase. In order to explain this phenomenon, KEGG enrichment analysis was used for the differential genes of cell clusters in the G1 phase (**Figure 4F**). Moreover, a PPI network was generated with the initial biomarkers (**Figure 4G**). As a result, the G1 phase and the process of glutamine metabolism were regulated by TP53.

### The landscape of genomic alterations between different risk cohorts and CAD is important to patients with IDH1 mutant glioma

**Figure 5:**
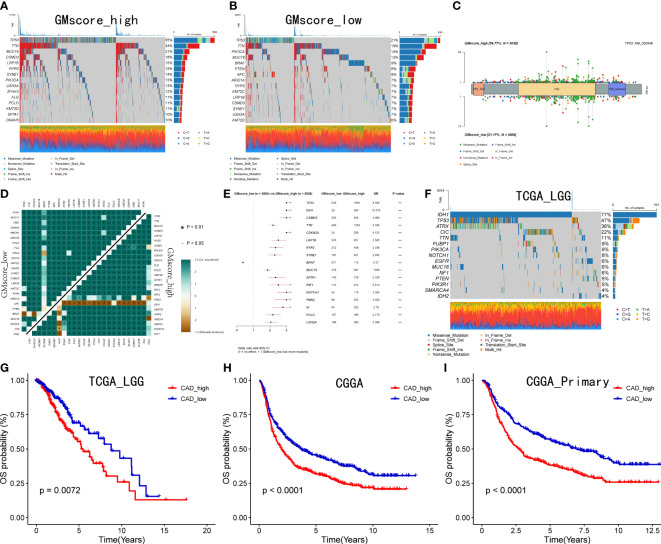
Genomic alterations between two risk groups and CAD is crucial to patients with IDH1 mutant glioma. **(A, B)** Top 15 most frequently mutated genes exhibited in two risk cohorts. **(C)** A lollipop plot showed the different spots of TP53 between the two risk cohorts. **(D)** Exclusive mutations related to IDH1 were observed in the GMscore-high cohort. **(E)** TP53 ranked first, and IDH1 owned the highest OR among all mutated genes ***p < 0.001. **(F)** Top 15 frequently mutated genes were illustrated in the LGG cohort. **(G–I)** Correlation between the expression of CAD and survival in TCGA LGG cohort, CGGA cohort, and patients with a primary tumor in CGGA cohort.

Since the development of cancer is often accompanied by alterations in the genome, we identified the top 15 genes with the most frequent mutations in various risk groups, respectively (**Figures 5A, B**). As shown in the oncoplots, TP53 occupied the first position among the genes both in the GMscore-high and -low cohorts. A lollipop plot illustrated the different spots of mutation in TP53, while more frequency and locations of mutations were defined in the GMscore-high cohort compared to the -low cohort (**Figure 5C**). Moreover, we compared the cumulative mutated proportion of classic carcinogenic pathways (**Supplementary Figure 4A**), which showed that the TP53 signaling pathway ranked first among all variables. Baslan et al. (38) demonstrated that the occurrence and development of cancer relied on an ordered determined genome evolution caused by the accumulation of TP53 inactivation. Combining the results in this study, we hypothesized that the cells lost precise regulation of the cell cycle and glutamine metabolism due to TP53 mutations. Moreover, the GMscore-high cohort, with frequent mutations of TP53, exhibited a poor prognosis. Then, we found some key genes that might be beneficial to clinical decisions and appropriate drug choices (**Supplementary Figure 4B).** Subsequently, co-occurrence and mutually exclusive mutations have been compared between the two risk groups, and a distinctive exclusion of IDH1 was observed (**Figure 5D**). Simultaneously, the forest plot showed that IDH1 owns the highest OR among all mutated genes (**Figure 5E**). Considering these results might account for the IDH1 mutation occurring in glioma (39), we analyzed IDH1 in specific tumors, including LGG and GBM in the TCGA cohort (**Figure 5F**, **Supplementary Figure 4D**); IDH1 exhibited the highest mutation frequency in TCGA LGG cohort (77%). Additionally, exclusive mutations of IDH1 were plotted (**Supplementary Figure 4C**). Two recent studies (40, 41) revealed that IDH1-mutant glioma cells are hypersensitive to drugs targeting enzymes in the *de novo* pyrimidine nucleotide synthesis pathway. As a major material in the process of synthesizing pyrimidine, glutamine is catalyzed by carbamoyl-phosphate synthetase 2, aspartate transcarbamylase, dihydroorotase (CAD) to supply nitrogen to the pyrimidine, which has become a key step in the synthesis of pyrimidine. Our data also presented that the expression level of CAD decreased evidently after inhibiting the glutamine metabolism (**Supplementary Figure 4E**). We then explored the correlation between the expression CAD and prognosis based on the median value of the different expression levels of CAD in the LGG and GBM cohorts from the TCGA database. Consequently, a significantly poor prognosis was observed in patients with high CAD expression in the LGG cohort (p<0.05), which was accompanied by the highest mutation frequency of IDH1 (**Figure 5G**). In the GBM cohort, which did not present mutated IDH1, no differences were observed between the expression level of CAD and prognosis (p>0.05, **Supplementary Figure 4F**). To validate the conclusion further, we retrieved the RNA-sequence data of patients with glioma in CGGA, showing that high expression of CAD had a poor prognosis (**Figure 5H, I**).

### Different immune characteristics between the GMscore-high and -low cohorts

**Figure 6:**
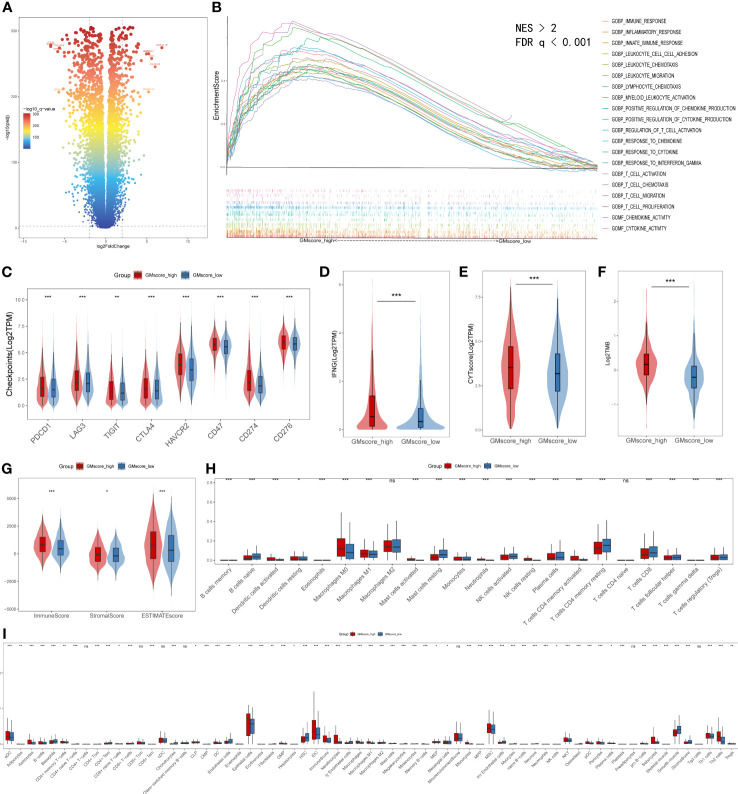
Immune characteristics between the GMscore-high and GMscore-low cohorts. **(A)** Differential genes between the two cohorts are shown in the volcano plot. **(B)** GSEA illustrated that the upregulated genes in the GMscore-high cohort were enriched in many immune-related pathways. GSEA, gene set enrichment analysis; NES, normalized enrichment scale. **(C)** The representative immune checkpoints, including PDCD1, LAG3, TIGIT, CTLA, CD47, CD274, and CD276, were significantly elevated in the GMscore-high cohort. **(D–G)** The GMscore-high cohort was characterized by significantly high INFG expression, CYT score, TMB, immune score, stromal score, and ESTIMATE score. **(H, I)** CIBERSORT and xCell algorithm qualified 28 and 67 types of immune cells between the two cohorts, respectively, and high immune infiltration was observed in the GMscore-high cohort. *p < 0.05; **p < 0.01; ***p < 0.001.

Regarding the immune infiltration between two risk cohorts, a series of bioinformatic methods were conducted to evaluate the immune landscape. Together, a threshold with FDR q<0.0001 and |log2FoldChange|≥2 defined 252 upregulated and 667 downregulated genes in the GMscore-high cohort (**Figure 6A**). GSEA analysis was conducted based on the differential genes, and the results showed that the upregulated genes in the GMscore-high cohort were enriched in many pathways that were related to immunity with a threshold of FDR q<0.001 and normalized enrichment score (NES)>2 (**Figure 6B**). The cytokine activity pathway ranked first with NES=2.47 (**Supplementary Figure 5A**). Additionally, we observed IL-10 as a pivotal regulator in cytokine activity *via* a PPI network (**Supplementary Figure 5B**), and the expression level of IL-10 elevated significantly in the GMscore-high cohort (**Supplementary Figure 5C**). These results indicated a high immune infiltration in the GMscore-high cohort. The latest findings reported that elevated IL-10 was closely associated with exhausting CD8+ T cells (42–44). Through measuring the number of CD8+ T cells of each patient by the TIMER algorithm, we found that CD8+ T cells decreased in the GMscore-high cohort (**Supplementary Figure 5D**). Next, we compared the common immune checkpoints, including PDCD1 (also known as PD-1), LAG3, TIGIT, CTLA4, HAVCR2, CD47, CD274 (also known as PD-L1), CD276, and other indicators, such as INFG, CYT score, and TMB. All these features were elevated in the GMscore-high cohort significantly (p<0.05, **Figures 6C–F**), which was consistent with a previous report (45). In addition, these results might indicate there was a better therapeutic response in the GMscore-high cohort. As many scientists had confirmed that the effect of glutamine on T cells depends on the cell type and state (23, 25, 45), to analyze different infiltration of other cells in the tumor microenvironment, three algorithms (ESTIMATE, CIBERSORT, and xCell) were used to measure the absolute and relative abundance of immune cells between the different cohorts. ESTIMATE demonstrated that the GMscore-high cohort was characterized by significantly high immune, stromal, and estimate scores and low tumor purity (**Figure 6G**, **Supplementary Figure 5E**). CIBERSORT was applied to evaluate the relative abundance of various immune cells, and the M2 macrophage was maximal (**Figure 6H**, **Supplementary Figure 5F**). The results of CIBERSORT and xCell (**Figure 6I**) are consistent with TIMER that CD8+ T cells decreased evidently in the GMscore-high cohort. And we found that many different types of cells were elevated in the GMscore-high cohort. Besides, we proved that the GMscore-high cohort, which with lower glutamine metabolic, presented a higher number of dendritic cells (DCs) as reported (46). Our data also matched a well-known report that IL-10 may play a vital role in the antitumor effect through DCs (47). In addition to DCs, the number of other immune cells like Th1 cells was up-regulated in the GMscore-high cohort.

### Sensitivity predictions of anti-cancer drugs and immunotherapy

**Figure 7:**
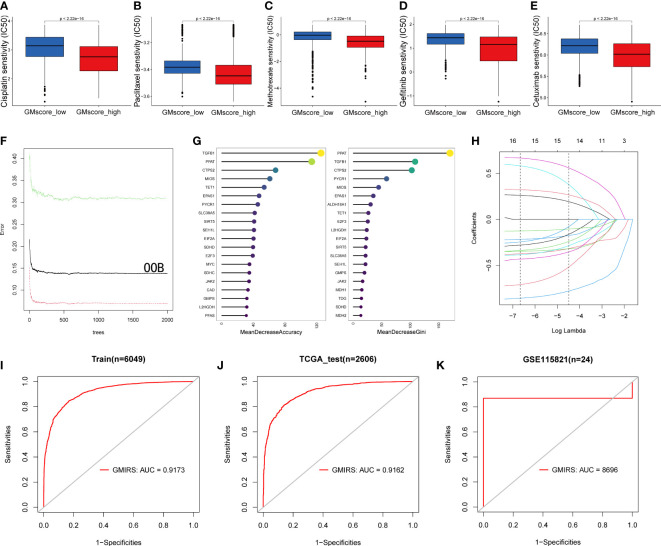
Impact of glutamine metabolism on anti-cancer drugs and immunotherapy. **(A–E)** Predictions of ICD50 values of the commonly used chemotherapy drugs (Cisplatin, Paclitaxel, and Methotrexate) and EGFR-targeted drugs (Gefitinib and Cetuximab) were significantly low in the GMscore-high cohort. **(F)** A random forest algorithm was applied to screen for the significant candidates related to the response of immunotherapy. **(G)** 15 genes were overlapped in the two ranking methods. **(H)** LASSO logistic regression analysis was used to construct a robust signature to predict the immunotherapy response. **(I–J)** GMIRS exhibited an AUC of 0.9173 and 0.9162 in the TCGA training and test cohorts, respectively. **(K)** GMIRS could describe the responses (AUC=0.8696) in patients treated with anti-PD1.

Based on the results before, we characterized the GMscore-high cohort with a lower metabolic level of glutamine. Some studies (48–50) pointed out that the endogenous nucleophile glutathione (GSH) could bind covalently with cisplatin, which may contribute to cisplatin resistance. This hinted to us that there might be a better therapeutic response of chemotherapeutic drugs in the GMscore-high cohort. Next, we investigated the differential sensitivities of commonly used chemotherapeutic drugs, including Cisplatin, Paclitaxel, and Methotrexate, and target EGFR drugs, such as Gefitinib and Cetuximab in each risk cohort with R package “pRRophetic”. (51). As we expected, the estimated IC50 value was significantly elevated in the GMscore-low cohort, indicating that the GMscore-high cohort might provide an improved outcome (**Figures 7A–E**). Nevertheless, our aforementioned data (**Figure 2A**) suggested that the malignancy of tumors might be negatively correlated with their mean GMscore. To confirm this hypothesis, data collected from the United States, the United Kingdom, and China (52) were further analyzed in order to avoid the errors caused by geographical, medical technology, and dietaries. The results suggested a significant negative correlation between the patients’ average five-year survival rate and GMscore (the United States: R=-0.77, p=0.0014; the United Kingdom: R=-0.76, p=0.0015; China: R=-0.71, p=0.0064, **Supplementary Figures 6A-C**). Sankey diagram showed the source of tumor types for patients in different risk cohorts (**Supplementary Figure 6D**). In order to improve the predictions of immunotherapy response in patients with different glutamine metabolism characteristics, we developed a brand new signature named glutamine metabolism immunotherapy response score (GMIRS) based on the initial biomarkers. As a result, the 118 genes with two optimal parameters (mtry=40, ntree=2000, **Figure 7F**) were ranked by two methods in the random forest analysis (**Figure 7G**). Then, the 16 overlapped candidates were input into LASSO regulation analysis (**Figure 7H**), and ten-fold cross-validation was conducted to overcome the over-fitting effect (**Supplementary Figure 6E**). Finally, 15 genes (*SLC38A5*, *CTPS2*, *SEH1L*, *L2HGDH*, *JAK2*, *TGFB1*, *E2F3*, *EPAS1*, *SIRT5*, *PPAT*, *TET1*, *EIF2A*, *MIOS*, *PYCR1*, *SDHD*) were selected to construct the predictive model. ROC analysis was applied to validate the accuracy, following which we observed that the response of ICB therapy could be predicted in the TCGA training and testing cohorts (GMIRS=0.9173, GMIRS=0.9162, **Figure 7I, J**). Additionally, RNA sequence data of 24 patients who received anti-PD1 therapy from GEO was used for further validation, and the results exhibited a high GMIRS with 0.8696, as expected (**Figure 7K**).

## Discussion

Herein, we generated a brand-new gene signature to guide the clinical decision-making of patients. A recent study (7) showed that high glutamine metabolism is a significant feature of tumor cells. The specific demand of tumor cells for glutamine makes it possible to develop new clinical predictions, treatment plans, and imaging strategies. Next, we used relevant genes involved in glutamine metabolism and regulation processes as the initial biomarkers. Finally, 21 genes related to glutamine metabolism (*CPS1*, *ME1*, *ASNS*, *SLC1A3*, *DEPDC5*, *SLC38A7*, *SLC7A5*, *TGFB1*, *MAPK8*, *WDR24*, *PSAT1*, *EGLN1*, *SDHC*, *GLUD1*, *SLC7A11*, *CXCL8*, *BCL2*, SLC25A22, *SHMT2*, *GFPT1*, and *SDHD*) were selected by machine learning to construct a prognostic gene signature related to glutamine metabolism. Among these, SLC38A7 and SLC7A5 are proteins used for glutamine transportation, GLUD1 and PSAT1 are necessary for glutamine catabolism, and some key enzymes (CPS1 and ASNS) involved in the biosynthesis of glutamine and its metabolites participated in the construction of this model. We divided the patients into two metabolic risk groups using the median value of signatures. After validating with significant clinical data, patients with low-risk scores showed improved prognoses. Furthermore, by combining a series of clinical features, we could accurately predict the OS of patients.

The unique metabolism of tumor cells often drives the development of other malignant characteristics. In the correlation analyses between glutamine metabolism and hallmarks of malignant tumors, a correlation was established between glutamine metabolism and cell cycle progression. Accumulating evidence (53) indicated that glutamine deprivation causes cell stagnation in the S phase. According to the current results, this phenomenon could be attributed to the lack of energy and raw materials in the biosynthesis of tumor cells during the S phase. Based on recent studies, we observed specific expression of glutamine metabolism-related genes in 1067 cells during the G1 (346 cells), S (334 cells), and G2M (387 cells) periods, respectively. We found that genes involved in glutamine uptake and catabolism into the TCA were highly expressed at the beginning of the G1 phase, while genes that used glutamine as a substrate to participate in biosynthetic steps were enriched in the S phase. RNA-sequence data from mice treated by inhibitor of glutamine metabolism confirmed that the expression level of these genes decreased indeed, while the change *in vivo* would be discussed in future. To further explore the reason for this phenomenon, we performed an enrichment analysis based on cycle-specific differential genes. The results showed that the G1 phase was regulated by the P53 signaling pathway, as described previously. The analysis of the metabolic regulation of glutamine revealed that the accumulation of *TP53* gene inactivation might be the link between glutamine and G1 phase regulation during the development of tumor cells. Nonetheless, the specific mechanism has not yet been clarified in our study, which will be proved in future experiments.

A recent study (54) found that the survival of IDH1 mutated glioma cells was dependent on the *de novo* synthesis of pyrimidine. The specific inhibition on DHODH, the key enzyme of pyrimidine synthesis, could have a very good effect on the treatment of patients with IDH1 mutant glioma. As a major enzyme in its upstream, CAD plays a key role in glutamine participation in pyrimidine synthesis. The comparison of the prognosis of patients with different expression levels implied that the use of targeted inhibitors of glutamine-related genes involved in CAD and its upstream could be an optimal treatment for IDH mutant glioma patients.

Based on the impact of glutamine metabolism on the immune microenvironment mentioned by Vander et al. (46, 55), we compared the differences in immune infiltration among patients in different risk groups. The high-risk scores were accompanied by high immune cell infiltration and activation of immune pathways. Among them, the cytokine activation pathway ranks first with NES=2.47. We identified IL-10 as the pivotal regulator in cytokine activity, and its level was upregulated significantly in the GMscore-high cohort. As an immunosuppressive factor, IL-10 is thought to be associated with exhausting CD8+ T cells (42–44). Besides, Leone RD et al. (56) showed that varied glutamine metabolic states can shape the TME into different immune landscapes. They proved that glutamine was vital for proliferating CD8+ T cells, and lacking glutamine can accelerate the depletion of CD8+ T cells and exhibit a higher expression level of immunosuppressive molecules such as PD-1 and LAG-3, which is constant with our result. Intriguingly, some studies (47, 57) illustrated that a half-life-extended IL-10–Fc can expand terminally exhausted CD8+ tumor-infiltrating lymphocytes (TILs) directly, which means IL-10 may function positively in the anti-tumor process. This might be the reason why DCs was up-regulated in the GMscore-high cohort with elevated IL-10. In addition to DCs, the other immune cells like Th1 cells, which recruit and activate macrophages and cytotoxic T cells mainly by expressing CD40L and cytokines like INFG and IL-2, also elevated in GMscore-high group (58). This suggested an up-regulation of macrophages and type II interferon (IFN) response. Consequently, all these conclusions indicated that the gene signature constructed in this study might be effective in guiding clinical treatment.

Our data proved that lower drug resistance and higher immune infiltration indicated better therapeutic response in the GMscore-high cohort. The comparison of the sensitivity of chemotherapy drugs and targeted drugs of patients in different risk groups revealed that the results did meet our expectations. As we mentioned above, GMscore has different distribution characteristics among tumors, and GMscore is normally elevated in some neoplasms of high malignancy such as glioma and pancreatic cancer while shows lower levels in some low malignancy tumors such as thyroid cancer, breast cancer, and prostate cancer. And We found that the majority of patients in the high-risk cohort were from highly malignant tumors. Tumors with high malignancy are characterized by recurrence and deterioration easily, directly leading to shorter OS in patients in the GMscore-high cohort. In addition, we found that the CD8+ T cell number in the GMscore-high group was significantly lower than that in the GMscore-low cohort, which may also result in the shorter OS of patients in the GMscore-high cohort. Furthermore, gene signatures derived from glutamine metabolism genes could predict the patient’s response to immunotherapy. These results guided the clinical medication of patients.

Taken together, glutamine metabolism is closely related to several malignant features of tumors, especially cell cycle disorder. Various metabolic levels predict different prognoses of patients. Various metabolic levels are observed under different immune landscapes. Therefore, the unique metabolic characteristics of patients may guide the personalized treatment of patients.

## Data availability statement

The original contributions presented in the study are included in the article/**Supplementary Material**. Further inquiries can be directed to the corresponding authors.

## Author contributions

Research design: ZL, ML and QS. Data collection: FL, SH, SZ, and YY. Data analysis: FL, SH and SZ. Manuscript preparation: BX, JW, SH and ZL. Chart preparation: YY, ZN, FL and SH. Revisions: ZL, ML and QS. Technical support: JW. All authors contributed to the article and approved the submitted version. All authors are also responsible for the manuscript content.

## Supplementary material

Supplementary Figure 1 Lower GMscore had an OS probability in many cohorts among various databases. **(A–D)** Kaplan–Meier analysis was used to validate patients with low GMscore who had improved prognoses in KIRC, LUAD, MESO, and UCEC cohorts from TCGA. **(E–H)** Kaplan-Meier analysis was used to validate patients with low GMscore with better prognoses in the four individual cohorts from GEO. **(I, J)**Kaplan-Meier analysis was used to validate the patients with low GMscore who had better prognoses in CGGA cohorts with 325 and 693 patients, respectively. **(K)** Significantly difference of baseline of the GMscores was observed among RNA-sequence data retrieved from TCGA and CGGA.

Supplementary Figure 2 Significant correlations between glutamine metabolism and hallmarks of the malignant tumor. **(A)** Significant correlations between glutamine metabolism and cell cycle progression among 32 types of cancer. **(B)** Top 8 significant correlations between glutamine metabolism and EMT. **(C)** Top 8 significant correlations between glutamine metabolism and immune infiltration. **(D)** Top 8 significant correlations between glutamine metabolism and angiogenesis. **(E)** Top 8 significant correlations between glutamine metabolism and inflammation.

Supplementary Figure 3 GMscore was significantly correlated with cell cycle progression. **(A)** Distribution of three clusters in pseudo time trajectory analysis. **(B)** Pseudo time trajectory analysis divided the cells into three clusters by state. **(C–F)** The expression of critical genes in charge of glutamine metabolism in different types of cells. **(G)** The expression level of critical genes decreased after inhibiting glutamine metabolism.

Supplementary Figure 4 Genomic alterations between two risk groups and CAD is crucial to patients with IDH1 mutant glioma. **(A)** A cumulative mutated proportion of classic carcinogenic pathways between different risk cohorts. **(B)** The possible genes that could be used to guide clinical decisions. **(C)** Co-occurrence and exclusive mutations in the TCGA LGG cohort. **(D)** The top 15 frequently mutated genes were illustrated in GBM cohorts. **(E)** The expression level of CAD decreased after inhibiting glutamine metabolism. **(F)** No significance (p>0.05) was detected in the prognosis based on the expression level of CAD in the GBM cohort.

Supplementary Figure 5 Different immune characteristics between the GMscore-high and -low cohorts. **(A)** The cytokine activity pathway ranked first in the GSEA with an NES of 2.47. **(B)** A PPI network was constructed, which revealed that the most important gene in the cytokine activity pathway is IL-10. **(C)** The expression level of IL-10 elevated in the GM-high cohort. **(D)** TIMER showed lower CD8+ T cells infiltration in GMscore-high cohort. **(E)** The GMscore-high cohort was characterized by significantly low tumor purity. **(F)** CIBERSORT algorithm was used to reveal that macrophage M2 accounts for the highest proportion among all the immune cells.

Supplementary Figure 6 Impact of glutamine metabolism on anti-cancer drugs and immunotherapy. **(A–C)** Negative correlation between the average five-year survival rate of each tumor and its matched GMscore. **(D)** Sankey diagram showed the source of tumor types for patients in different risk cohorts. **(E)** The optimal λ was identified with ten-fold-cross-validation.

Supplementary Table 1 Initial biomarkers of glutamine metabolism.
