# Supplementary figures and images for: Turbulence of glutamine metabolism in pan-cancer prognosis and immune microenvironment
Source: Front Oncol. 2022 Dec 7;12:1064127. doi: 10.3389/fonc.2022.1064127 (PMC9769123; doi:10.3389/fonc.2022.1064127)

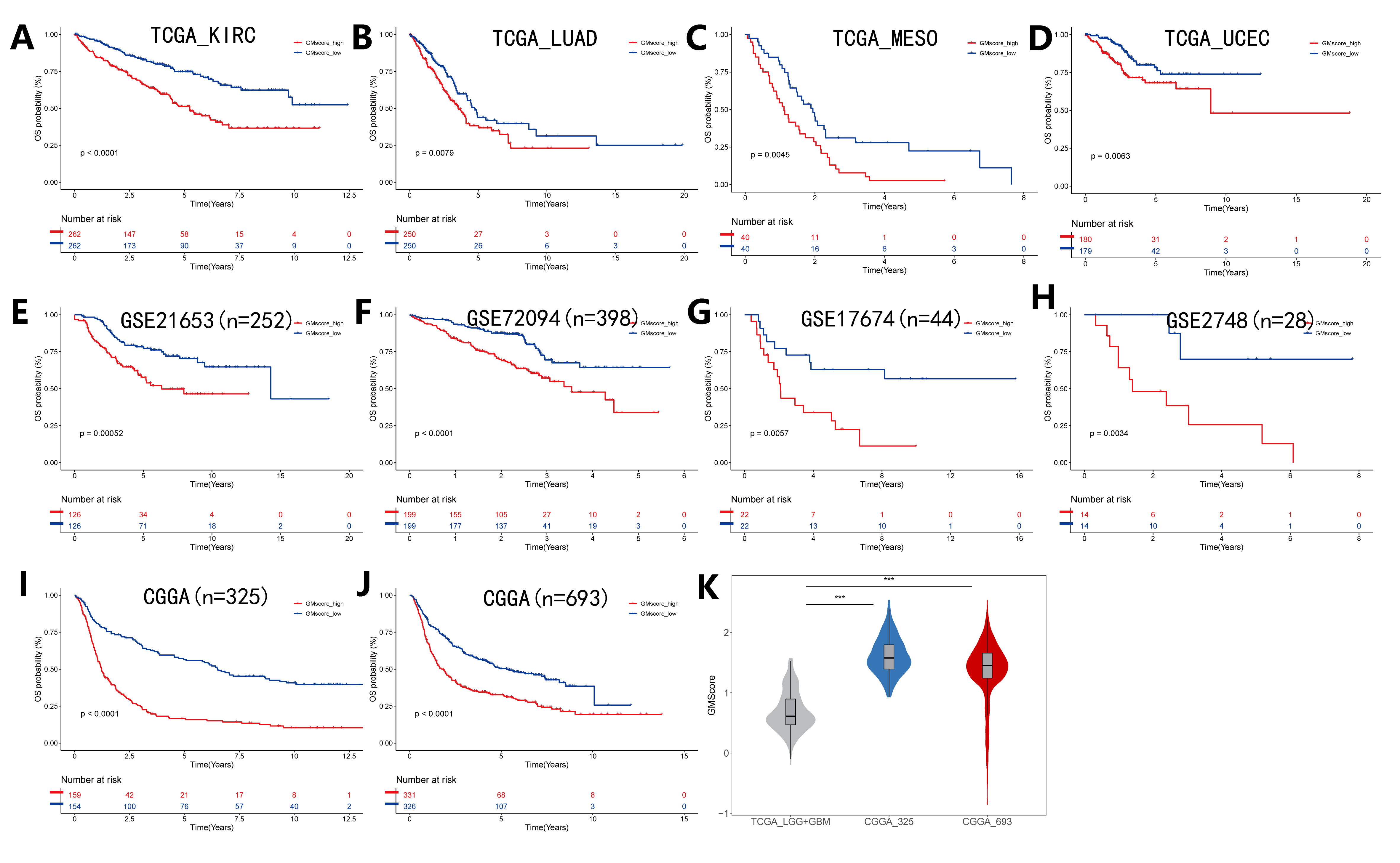

Supplement: Supplementary Figure 1 — Lower GMscore had an OS probability in many cohorts among various databases. (A–D) Kaplan–Meier analysis was used to validate patients with low GMscore who had improved prognoses in KIRC, LUAD, MESO, and UCEC cohorts from TCGA. (E–H) Kaplan-Meier analysis was used to validate patients with low GMscore with better prognoses in the four individual cohorts from GEO. (I, J)Kaplan-Meier analysis was used to validate the patients with low GMscore who had better prognoses in CGGA cohorts with 325 and 693 patients, respectively. (K) Significantly difference of baseline of the GMscores was observed among RNA-sequence data retrieved from TCGA and CGGA. [file Image_1.tif]

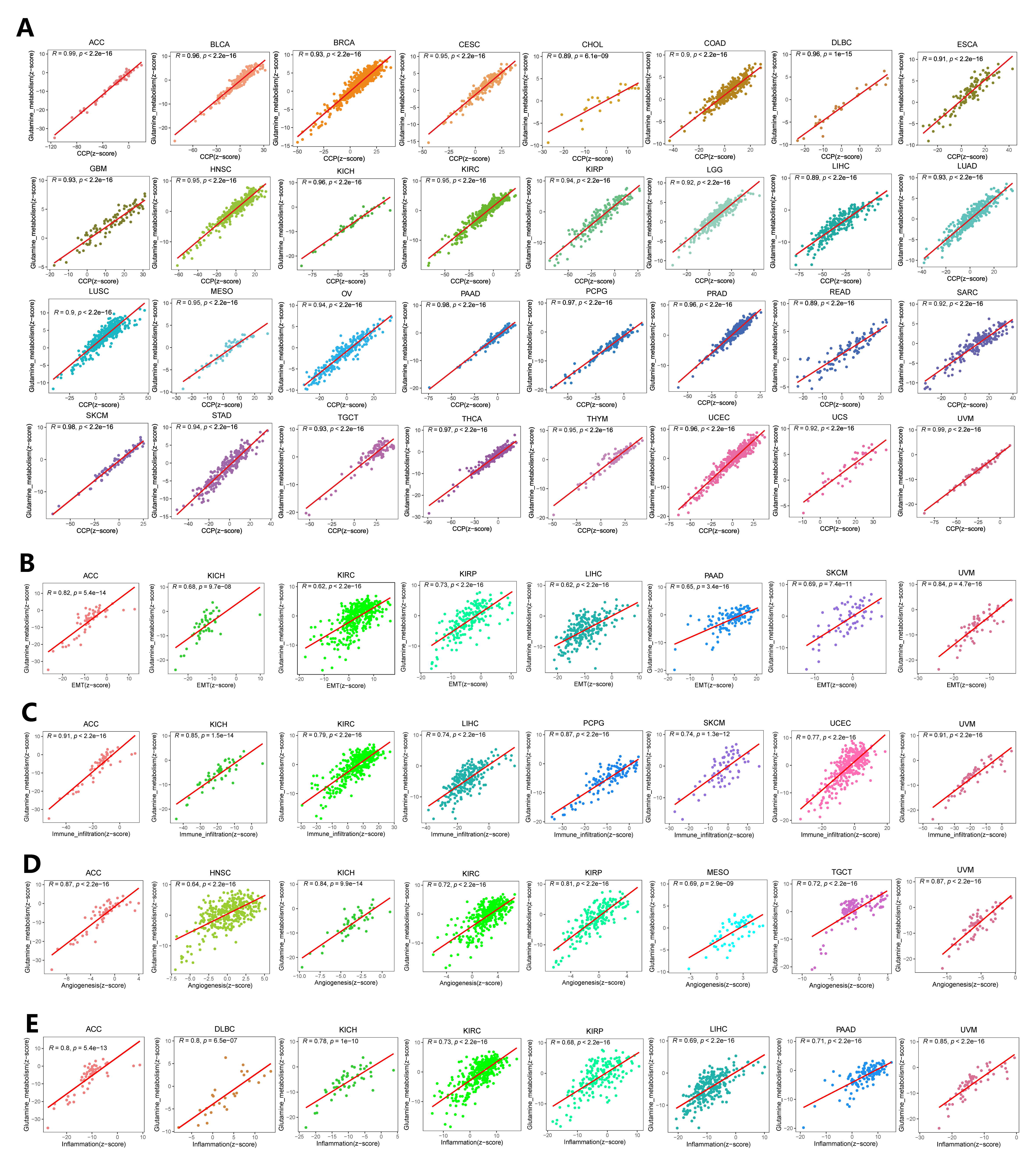

Supplement: Supplementary Figure 2 — Significant correlations between glutamine metabolism and hallmarks of the malignant tumor. (A) Significant correlations between glutamine metabolism and cell cycle progression among 32 types of cancer. (B) Top 8 significant correlations between glutamine metabolism and EMT. (C) Top 8 significant correlations between glutamine metabolism and immune infiltration. (D) Top 8 significant correlations between glutamine metabolism and angiogenesis. (E) Top 8 significant correlations between glutamine metabolism and inflammation. [file Image_2.tif]

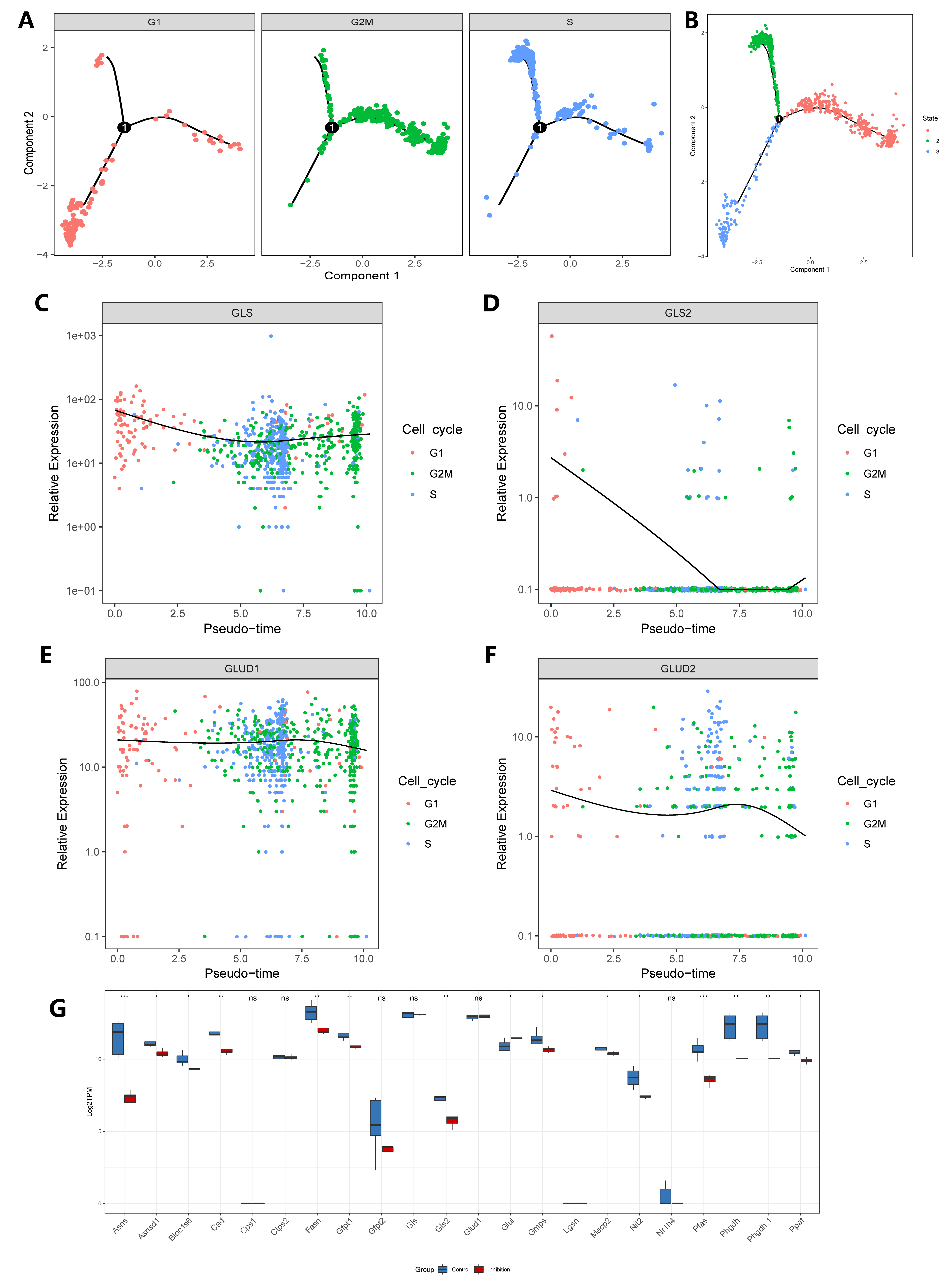

Supplement: Supplementary Figure 3 — GMscore was significantly correlated with cell cycle progression. (A) Distribution of three clusters in pseudo time trajectory analysis. (B) Pseudo time trajectory analysis divided the cells into three clusters by state. (C–F) The expression of critical genes in charge of glutamine metabolism in different types of cells. (G) The expression level of critical genes decreased after inhibiting glutamine metabolism. [file Image_3.tif]

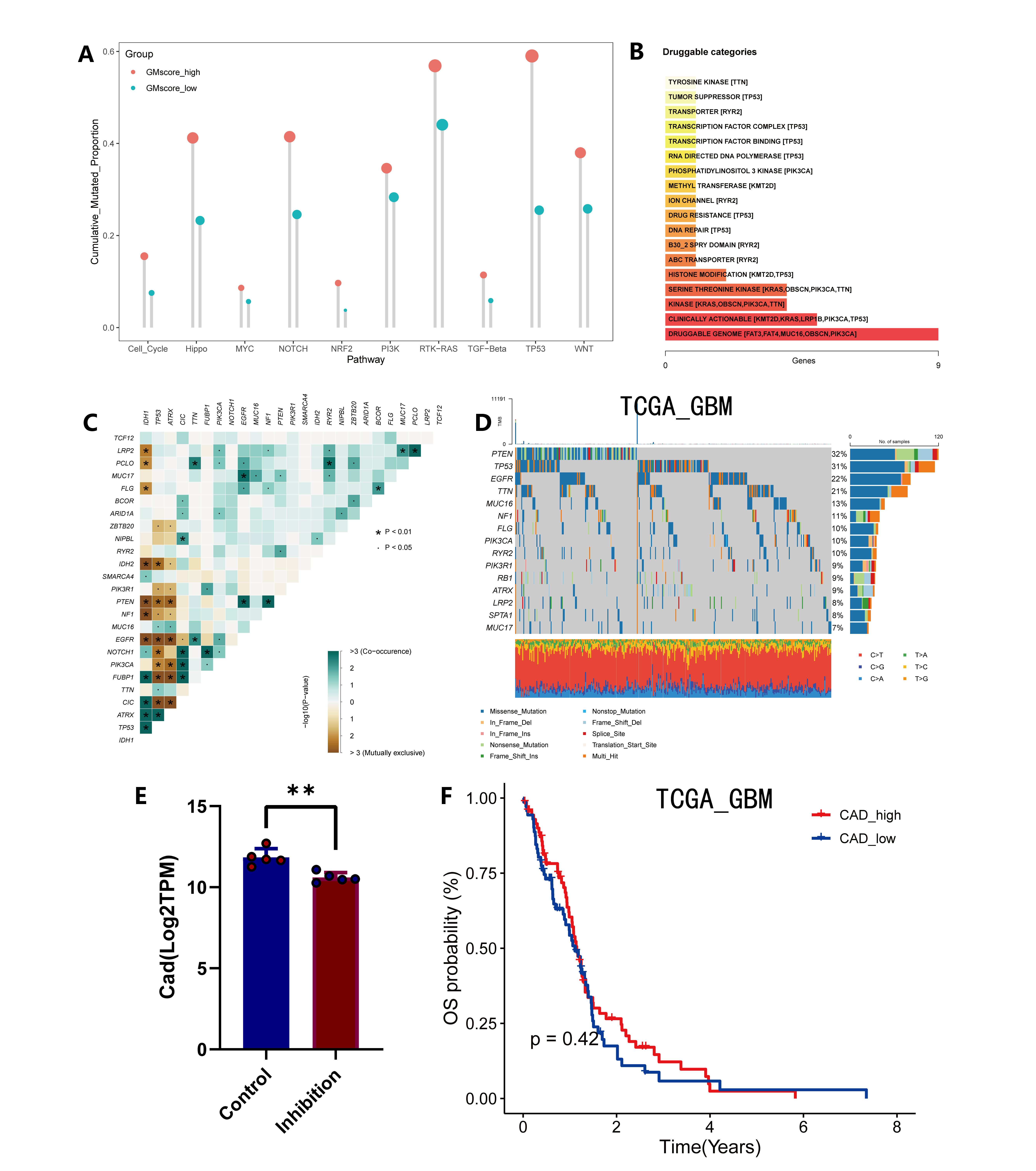

Supplement: Supplementary Figure 4 — Genomic alterations between two risk groups and CAD is crucial to patients with IDH1 mutant glioma. (A) A cumulative mutated proportion of classic carcinogenic pathways between different risk cohorts. (B) The possible genes that could be used to guide clinical decisions. (C) Co-occurrence and exclusive mutations in the TCGA LGG cohort. (D) The top 15 frequently mutated genes were illustrated in GBM cohorts. (E) The expression level of CAD decreased after inhibiting glutamine metabolism. (F) No significance (p>0.05) was detected in the prognosis based on the expression level of CAD in the GBM cohort. [file Image_4.tif]

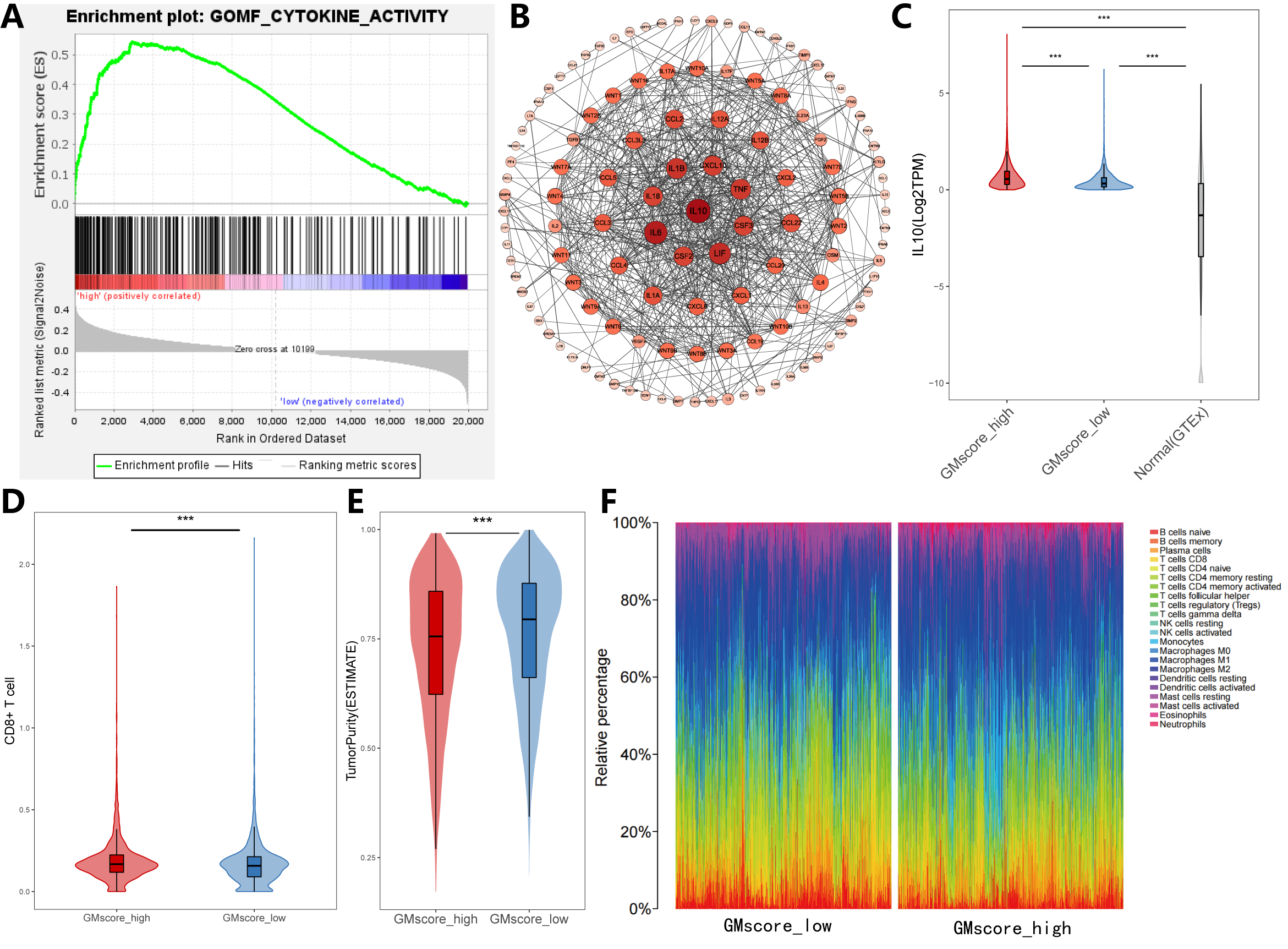

Supplement: Supplementary Figure 5 — Different immune characteristics between the GMscore-high and -low cohorts. (A) The cytokine activity pathway ranked first in the GSEA with an NES of 2.47. (B) A PPI network was constructed, which revealed that the most important gene in the cytokine activity pathway is IL-10. (C) The expression level of IL-10 elevated in the GM-high cohort. (D) TIMER showed lower CD8+ T cells infiltration in GMscore-high cohort. (E) The GMscore-high cohort was characterized by significantly low tumor purity. (F) CIBERSORT algorithm was used to reveal that macrophage M2 accounts for the highest proportion among all the immune cells. [file Image_5.tif]

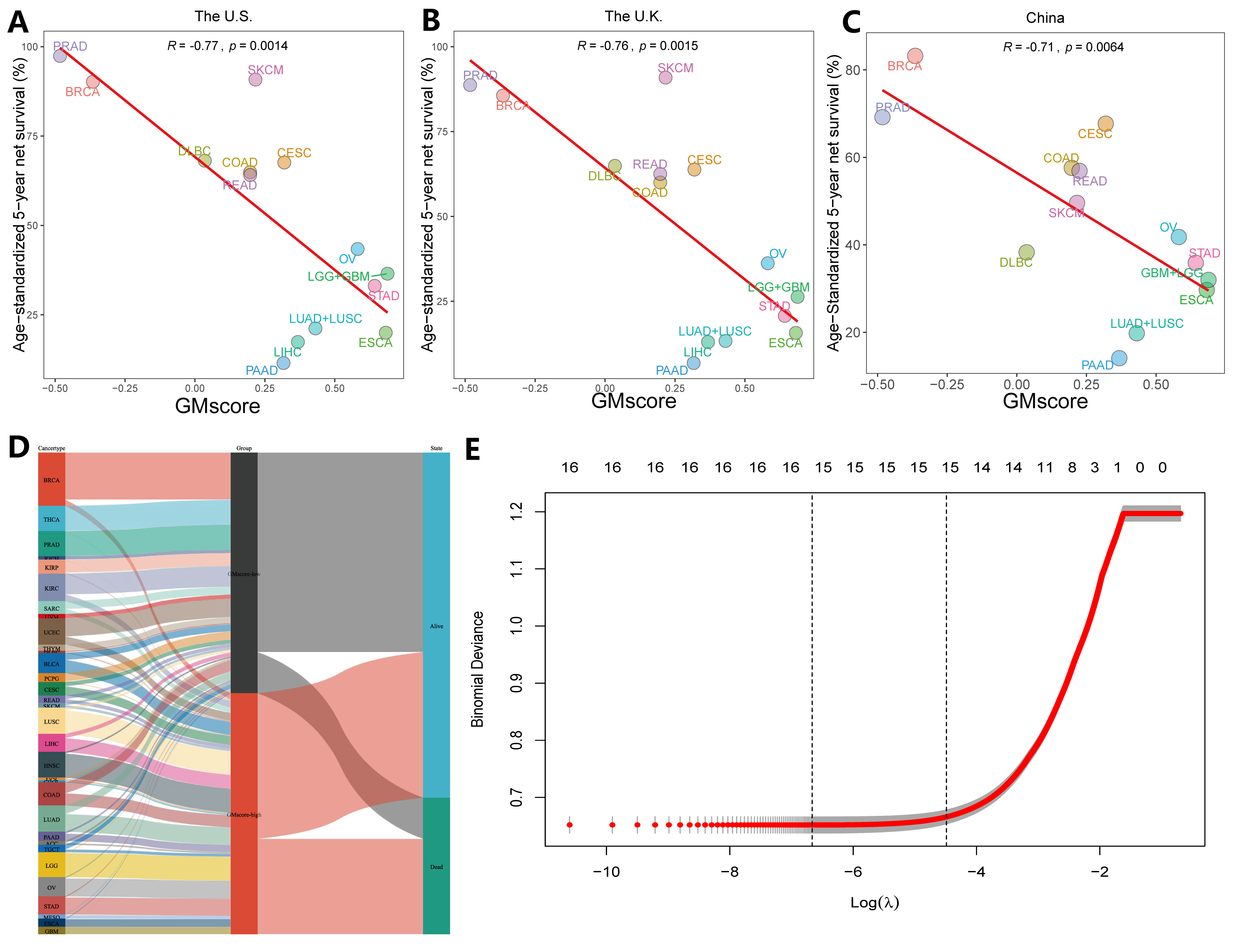

Supplement: Supplementary Figure 6 — Impact of glutamine metabolism on anti-cancer drugs and immunotherapy. (A–C) Negative correlation between the average five-year survival rate of each tumor and its matched GMscore. (D) Sankey diagram showed the source of tumor types for patients in different risk cohorts. (E) The optimal λ was identified with ten-fold-cross-validation. [file Image_6.tif]
